# In the Midst of Life

**Published:** 1888-08-11

**Authors:** 


### The Doctor's Pulpit.

### "IN THE MIDST OF LIFE."

"In the midst of life we are in death." These simple words of the Prayer-book have lately received impressive confirmation in the early death of one who was well known to some of the readers of this journal. He was not a public man, and those who did not know him can have no motive for tracing his identity The interest of his sudden and early death lies in the fact that he was one of a large class who slowly but surely kill themselves in mid-life by too great a devotion to secular duty. The members of his family, who will easily recognise the brief outlines here given, will not grudge the use which is made of his short life-story Briefly it was this: A young Scotchman, of good early education and habits, with absolutely no vices, came up to London a little more than twenty years ago to mend his fortunes. His fortunes were mended. He was a man of business capacity, of great industry, and of the strictest integrity. He fitted himself by the study of languages for important posts, and important posts were in due time assigned to him. He soon acquired a position which to some men would have been enough for ambition. It was not, however, enough for him. He had an iron will and a power of self-denial which were positively heroic, and he determined to compel a higher fortune. He stuck to his work, both in his office and at home, devoting his days to routine business and his evenings to study. He mastered languages, political economy, and geography. He gave some attention to other sciences and much to politics, and in every way strove to fit himself for higher things, if they should ever come naturally or be compelled to come in his way

But fortune will not always be compelled. Do what a man will, he sometimes fails of the goal of his ambition. He may "burn the midnight oil," give up sleep, and "live laborious days," and yet be disappointed. This man knew no failure of courage, no abatement of resolution. But though the spirit was willing with an unconquerable willingness, the flesh yielded before the fight was over. At the age of forty-four, with a previous illness of only a few weeks, an illness which consisted of brain prostration alone, he died suddenly of apoplexy.

His struggle with the world was a twenty years' tragedy. Early marriage, a large family, means for necessities but none for advancement, a passion for progress which no persuasion of friends or bodily pain and weakness could abate, a struggle carried on to the very last moment, and then collapse, hopelessness, death. Such is a portion of the life history of one man. How many similar stories could London tell in one year! How many could Manchester, could Glasgow, could Paris, could New York tell!

Well, then, the doctor, being a mortal man, with some of the natural characteristics of humanity, and some of the instincts of his life-saving trade about him, asks, Why should this be? Why is this tragedy going on every year, every week, every day, in ten thousand offices, pulpits, consulting-rooms, shops? Whose duty is it to make the inquiry and to demand an answer? Is it the preacher's, or the political economist's, or the statesman's, or the doctor's? Is it not any man's and every man's who has eyes to see, a mind to understand, and a pen to write? The doctor looks at it from his point of view, and he declares that this particular life has been flung away: flung away—partly by the rushing and grinding world which caught him in its whirlpool, partly by a false theory of life, and partly by an almost complete ignorance of physiology and the elementary conditions of healthy existence. Concerning the grinding world and its mad, resistless whirlpool, a man who desires to be "hard down" on practicalities will say very little. The restless multitude have always been more or less blind and mad. In old times a certain herd of swine, being possessed with devils, ran violently down a steep place into the sea, and were choked with the waters. M. Renan might deny that the record states a fact, but even he would admit that the story furnishes a most apt illustration of much of human life. Calm reason—where is it? Deliberate purpose—who possesses it? Patient continuance in well-doing, and contentment with the slow but sure rewards of such method of a life—who cares for these? The world is a gambler's maelstrom, and all but one in a million jump in and test their destiny. A grand result this of nearly nineteen centuries of Christianity, of a hundred years of progressive science, of multiplied universities, and costly school boards!

But these tragic deaths midway in the journey are often the result of a false theory of life. A boy, entering upon manhood, opens his eyes slowly to the doings of the great world. He cannot take into his mind any true conception of the whole play he sees before him. What strikes his imagination is that here and there one man stands prominently out from his fellows. He is a statesman, or a preacher, or a man of business, or a physician. Of him all men speak, by him some men set the watches of their opinions, his lead a multitude will follow like sheep. He is the man, thinks Inexperience. He is blessed, whatever others be. For him life is a success and a triumph. To be like him should be the one object of existence. Inexperience thereupon makes choice of the course which he thinks will best suit his own speed and wind, and off he starts to catch up and pass the leader. One in ten millions succeeds. What of the rest? This, of reaching the summit of human ambition at all hazards, is a false theory of life, and as irrational as it is false. The only true theory is for a man always to do the best and honestest that is in him, to take the reward which naturally falls to such doing, and to be content with it.

Multitudes fail, as did he whom we have quoted, from a want of knowledge of physiology and the elementary conditions of bodily health. A man will study how to get food for his body, how to procure clothing, how to raise a roof over his head, but he will not give a moment's attention to the body which is going to make use of all these things. He will labour to acquire knowledge, intellectual aptitudes, and technical skill; but he will not take one thought for the condition of that brain without which his knowledge and his aptitudes are as a tuneful organ with the player dead on his stool. Will men ever learn that it is only the organ which is in good repair and tune from which the noblest music can come? The very first and most indispensable of all conditions of perfect music is that the organ be in perfect tune. The simile is exact as applied to man. Health of brain and body are absolutely essential to the production of the best work of which he is capable. He who works when out of health, not being compelled to do so, thereby confesses, either that he is not clever enough to know that he is doing inferior work, or that he is so foolish as to think that the world will not know or care whether he is doing his best or not. Health! Health first of all, if a man is to fight his bravest and work his best. This is one of the most elementary of all the truths which science and experience alike teach. To despise this is like trying to make progress in arithmetic without first learning the multiplication table. For want of a deep and practical conviction of this elementary truth, the world's highway is strewn with the miserable wrecks of the living and with the bodies of the defeated dead.

